# Molecular characterization of the apoptosis-related *SH3RF1* and *SH3RF2* genes and their association with exercise performance in Arabian horses

**DOI:** 10.1186/s12917-018-1567-0

**Published:** 2018-08-14

**Authors:** K. Ropka-Molik, M. Stefaniuk-Szmukier, K. Piórkowska, T. Szmatoła, M. Bugno-Poniewierska

**Affiliations:** 1 Department of Animal Molecular Biology, National Research Institute of Animal Production, Kraków, Poland; 2 Department of Horse Breeding, Institute of Animal Science, the University of Agriculture in Cracow, Kraków, Poland; 3 Institute of Veterinary Sciences University of Agriculture in Krakow, Kraków, Poland; 4 Laboratory of Genomics, National Research Institute of Animal Production, Krakowska 1, 32-083 Balice, Poland

**Keywords:** POSH (gene), POSHER (gene), Arabian horses, Training, Apoptosis, Flat-racing, Gene expression

## Abstract

**Background:**

Apoptosis plays an important role in the regulation of healthy tissue growth and development as well as in controlling the maintenance of homeostasis in exercising muscles. During an intensive physical effort, the regulation of cell death by apoptosis results in the replacement of unaccustomed muscle cells by new cells that are better suited to exercise. The aim of this study was to determine the expression of two genes (*SH3FR1* and *SH3RF2*) that control apoptosis in muscle tissues during training periods characterized by different intensities. The gene expression levels were estimated using real-time PCR method in skeletal muscle biopsies collected from 15 Arabian horses (untrained, after an intense gallop phase, and at the end of the racing season). An association study was performed on 250 Arabian horses to assess the effect of the *SH3RF2:*c.796 T > C (p.Ser266Pro) variant on race performance traits in flat gallop-racing.

**Results:**

A gene expression analysis confirmed a significant decrease (*p* < 0.01) in the anti-apoptotic *SH3RF2* (*POSHER*) gene during training periods that differed in intensity. The highest *SH3RF2* expression level was detected in the muscles of untrained horses, whereas the lowest expression was identified at the end of the racing season in horses that were fully adapted to the exercise. A non-significant decrease in *SH3RF1* gene expression following the training periods was observed. Moreover, a serine substitution by proline at amino acid position 266 (*CC* genotype) was negatively associated with the probability of winning races, the number of races in which a horse occurred and the financial value of the prizes. Horses with the *TT* genotype achieved the highest financial benefits, both for total winnings and for winnings per race in which the horses participated.

**Conclusions:**

The present study showed the supposed regulation mechanism of exercise-induced apoptosis in horses at the molecular level. The identified *SH3RF2*: c.796 T > C missense variant was associated with selected racing performance traits, which is important information during the evaluation of horses’ exercise predisposition. The association results and frequencies of the *CT* and *TT* genotypes suggest the possibility of using *SH3RF2* variant in selection to improve the racing performance of Arabian horses.

**Electronic supplementary material:**

The online version of this article (10.1186/s12917-018-1567-0) contains supplementary material, which is available to authorized users.

## Background

Biological processes that occur in the body during exercise have been widely studied in terms of the physiological response and muscle adaptation to demand. Skeletal muscle adaptation during intensive training occurs through modification of the metabolic rate, the regulation of oxygen uptake, the production of lactate and the usage of energy from various sources, such as glycogen or fat [1]. Skeletal tissue remodelling is another adaptation mechanism related to exercise, fitness, and muscular strength [2]. Exercise can induce an increase in muscle cell size (hypertrophy) [3] and the number of fibres (hyperplasia) [4] or lead to the transformation of fibre types [5]. Furthermore, several reports have shown that one of the processes related to the adaptation to the training is apoptosis or programmed cell death [6, 7].

Apoptosis plays an important role in the regulation of healthy tissue growth and development, is critical during ageing and in some diseases, and controls the maintenance of homeostasis in the exercise muscle [6]. Research performed on rats has shown that physical activity is associated with the intensity of the apoptotic processes in both skeletal and cardiac muscles [8]. In thoroughbred horses, Boffi et al. [9] confirmed that the training induces apoptosis in skeletal muscle. The authors hypothesized that the regulation of cell death by apoptosis results from the replacement of unaccustomed muscle cells by new and stronger cells that are better suited to the exercise. In Arabian horses, a whole transcriptome analysis of muscle tissues showed a significant down-regulation of several genes during training periods of different intensities, but the expression of only one gene, *SH3 domain containing ring finger 2* (*SH3RF2* previously known as *POSHER*), was lower throughout all training points in untrained animals compared to fully exercise-adapted horses [10]. The comparison of muscle transcriptomes between horses in different training periods enabled the identification of differentially expressed genes that are likely related to an adaptation to exercise intensity [10]. The next stage of the research should be the verification of the selected gene’s association with the performance features via the identification of variants with a potential effect on gene expression as well as on protein activity and function.

*SH3RF2* encodes an E3 ubiquitin-protein ligase (SH3 Domain Containing Ring Finger 2) that, together with its homologue (POSH; SH3RF1), regulates the intensity of apoptotic processes via modification of JNK signalling [11]. The SH3RF2 protein is considered to be an anti-apoptotic factor, the overexpression of which leads to a decrease in apoptosis promotion [11]. On the other hand, the decrease of SH3RF2 content promotes stabilization of its homologue – SH3RF1 and, via activation of the JNK cascade induces apoptosis of multiple cell types [11]. The SH3RF1 as pro-apoptotic protein induces JNK activation and cell death and together with SH3RF2 protein determine a dynamic mechanism that regulates apoptosis in cells [11, 12]. The recent report showed also that SH3RF1 acts as a negative post-translational regulator of atypical cadherin 1 (FAT1) essential for controlling cell proliferation [13]. The aim of this study was to determine the exact expression level of both the *SH3RF1* and *SH3RF2* genes in muscle tissues during training periods characterized by different intensities. The second part of the presented research focused on estimating the possible effects of a variant within the *SH3RF2* (*POSHER) locus* on race performance traits in flat gallop-racing.

## Methods

### Gene expression analysis

The expression of two genes, *SH3RF1* and *SH3RF2*, in muscle were estimated (*m. gluteus medius*) from samples that were collected from 15 unrelated pure-bred Arabian horses: 5 untrained horses (2.5-year-old horses) and 3-year horses during a one-year training period preparing for flat races at two time points (after an intense gallop phase, *n* = 10; at the end of the racing season, *n* = 8). The exact training procedure and points of muscle collection were described previously [10]. Muscle biopsy was performed using a ProMag™ Ultra Automatic Biopsy Instrument (Argon Medical Devices, Inc., Creek Road, USA) with a 2-mm-diameter biopsy needle according to the procedure described by Stefaniuk et al. [14]. The experimental protocol was approved by the Animal Care and Use Committee of the Institute of Pharmacology, Polish Academy of Sciences in Cracow (no. 1173/2015). The horses’ owners gave consent to be part of this study and allowed for maintaining all research procedures on animals.

Total RNA was isolated using TriReagent, and the RNA quality and quantity were determined using a TapeStation 2200 instrument (Agilent Technologies, Warsaw, Poland) and RNA Screen Tape (Agilent Technologies). RNA samples (300 ng each) with RIN values above 8.5 were reverse transcribed to cDNA using the High-Capacity RNA-to-cDNA Kit (Applied Biosystems, Thermo Scientific, Waltham, Massachusetts, USA). The expression levels of both genes were detected on a Quant Studio 7 flex real-time PCR system (Applied Biosystems) using AmpliQ 5× HOT EvaGreen® qPCR Mix Plus (ROX) (Novazym, Poland) in 3 replicates for each sample. The primers for the targeted genes and endogenous controls (*GAPDH* and *B2M*) [15] were designed using Primer3 version 4.0.0 based on the Ensemble reference sequence (Additional file 1: Table S1). The primers for *SH3RF2* gene were not affected by the identified c.796 T > C missense variant.

The PCR efficiencies were estimated based on the standard curve method, and the normalization factor (NF) was calculated based on the geometric mean of the normalized quantity of the two endogenous genes (*GAPDH* and *B2M*). The relative quantities of the analysed genes were calculated using delta-delta Ct according to Pfaffl [16]. The differences between training periods in the expression levels were analysed using SAS software (SAS Institute, Cary, NC, v. 8.02, 2001). The normality of the distribution was tested using the Shapiro-Wilk test, and differences between the groups were analysed using the Kruskal–Wallis test.

### Association study of the SH3RF2 (POSHER) c.796 T > C missense variant and racing results

The association study was performed on 250 pure-bred Arabian horses, from which biological material was collected as blood or hair follicles. The analysed Arabian horses were the offspring of 93 stallions (an average of 2.68 individuals per father) and 206 mares (an average of 1.21 individuals per mother). All 3- to 5-year-old horses had taken part in flat races at distances ranging from 1400 to 3000 m. The racing performance traits considered were wins by taking 1st, 2nd or 3rd place and the total number of wins from 1st to 3rd places, the number of races in which the horse participated, and the financial benefits obtained by the horses (wins resulting in money).

Based on RNA-seq data previously obtained from Arabian horse blood and muscle transcriptome sequencing (GEO databases: GSE83404; GSE88951), all of the coding sequences and 3′ and 5’ UTR regions were screened to detect polymorphisms. DNA was isolated using the Sherlock AX DNA Isolation kit (A&A Biotechnology, Gdynia, Poland). Next, the PCR-RFLP method was applied to genotype the only identified mutation, the ENSECAT00000026355.1:c.796 T > C missense variant (ss#2137535205; rs396219497). A 479-bp fragment of the *SH3RF2* gene (F TTTTTAGCCCCTTTGGACCT; R GGTGCTGATCTCCACCATTT) was amplified using AmpliTaq Gold® 360 Master Mix (Thermo Scientific) according to the protocol with a 57 °C annealing temperature. The PCR products were digested using the BtsCI endonuclease and the obtained fragments were allele T - 289, 151 and 39 bp and allele C – 440 and 39 bp.

The associations between the identified mutations and racing results were estimated using the GLM procedure in R software (R version 3.4.4).

## Results and discussion

### Exercise-induce modifications of SH3RF1 and SH3RF2 expression

The exact mechanisms and effects of exercise-induced apoptosis on the maintenance of body homeostasis and adaptation to training are still unclear. Siu et al. [8] indicated that during exercise, cell death can be activated by reactive oxygen species. Oxidative stress can damage the mitochondria and affect mitochondrial-mediated apoptosis. On the other hand, apoptosis can be an important mechanism that protects muscles from damage and controls myoblast proliferation through muscle regeneration [6]. Phaneuf and Leeuwenburgh [7] hypothesized that training-induced apoptosis is a regulatory process that is intended to remove certain damaged cells without causing a distinct inflammatory response.

There is very little information on the occurrence of the apoptotic process in the muscles of exercising horses. Boffi et al. [9] inferred that apoptosis has an important role during muscle remodelling and fitness maintenance in thoroughbred horses. The authors confirmed the occurrence of the apoptotic process in *gluteus medius* muscles and found that it was related to the programmed death of cells unadapted to exercise conditions and their replacement by new cells that are more suited to the increased physical effort. Furthermore, our previous study showed exercise-induced de-regulation of gene expression related to programmed cell death of the genes *SH3RF2*, *BCL2*, and *ANKRD2* [10]. These findings confirm the association of the apoptotic processes during muscle adaptation to training.

**Fig. 1:**
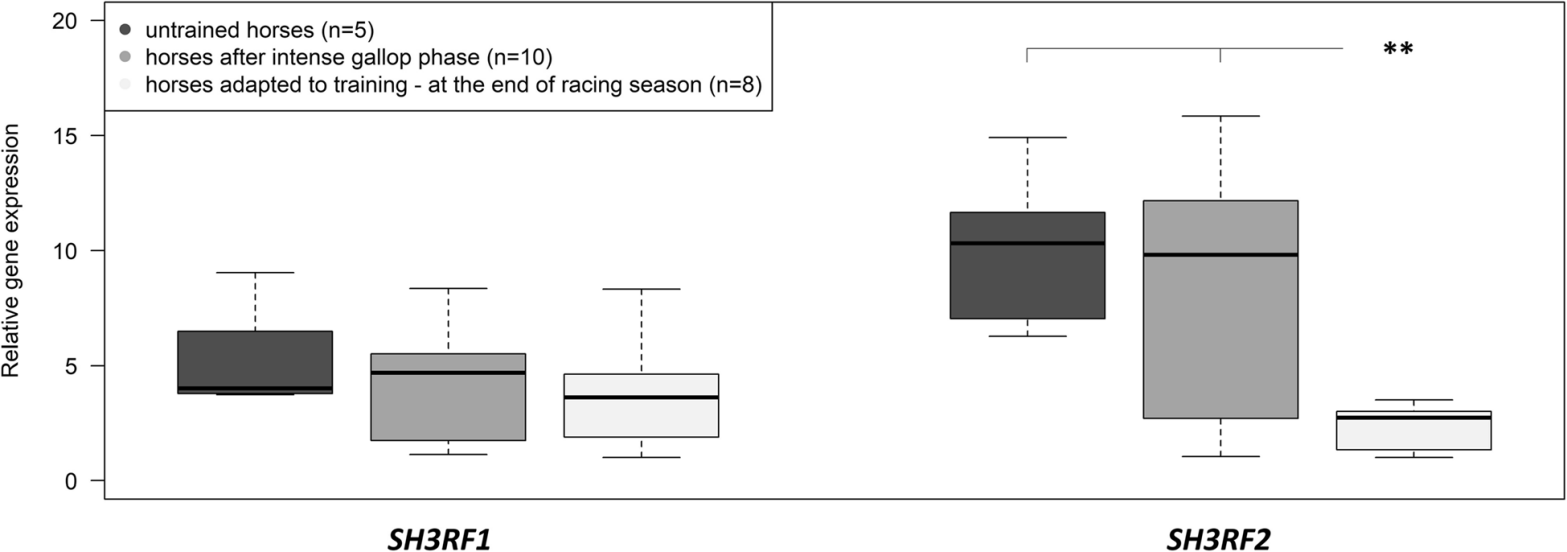
*SH3RF1* and *SH3RF2* gene expression profiles in muscle tissues (*m. gluteus medius*) of horses during a training regimen estimated based on two endogenous controls (*GAPDH and B2M genes*). Data are presented as the mean ± standard error; ** *p* < 0.01

The use of a precise, real-time PCR method allowed us to confirm the significant decrease (*p* < 0.01) of the anti-apoptotic *SH3RF2* gene in the muscle tissue of Arabian horses during training periods that differed in intensity. The highest *SH3RF2* expression level of the *SH3RF2* gene was detected in muscles of untrained horses, whereas the lowest expression was identified at the end of the racing season in horses that were fully adapted to exercise (Fig. 1). Our results also showed a decrease in *SH3RF1* gene expression following the training periods, but this difference was not significant (Fig. 1). A significant and more than 5-fold decrease of the anti-apoptotic *SH3RF2* gene in muscles adapted to training compared to untrained tissue might indicate that exercise-induced apoptosis can be a physiological response mechanism essential for fitness maintenance in horses. However, the association of SH3RF2 with adaptation to exercise should be confirmed at the protein level, which will provide full information about the regulation of an investigated anti-apoptotic factor during physiological effort. Boffi et al. [9] suggested that in thoroughbred horses, apoptosis can be a natural mechanism that regulates muscle strength as opposed to necrosis, which is evidence of overtraining.

It is assumed that exercise-induced apoptosis is promoted by the up-regulation of pro-apoptotic genes and/or the down-regulation of anti-apoptotic genes [6, 17]. The decrease in *SH3RF2* gene expression observed in the present study supports this thesis and suggests which molecular pathway might be involved in the regulation of apoptosis following exercise. The anti-apoptotic function of *SH3RF2* is based on *SH3RF1* degradation because it is a pro-apoptotic protein. On the other hand, a decrease in the SH3RF2 protein level in cells leads to SH3RF1 stabilization as a result of activation of the JNK signalling pathways and apoptosis [11]. Moreover, Kim et al. [18] showed that POSHER (SH3RF2) can control actin cytoskeleton modification by mediating PAK4 protein stability.

The interaction networks of the SH3RF2 protein created using the String Database based on *Equus caballus* [19] indicated that SH3RF2 is associated with growth hormone receptor (GHR), thyroid stimulating hormone receptor (TSHR) and programmed cell death 6 interacting protein (PDCD6IP) (Additional file 2: Figure S2B). Additionally, it has been experimentally determined that human SH3RF2 is related to SH3RF1 (POSH) [11] and protein phosphatase 1 (PPPC1A) [20] (Additional file 2: Figure S2A). Gesing et al. [21] confirmed that *Ghr*-null mice have a delayed ageing process and prolonged longevity. Moreover, in vitro studies showed that an increase in *TSHR* gene expression can lead to apoptosis activation [22]. Such findings suggest that *SH3RF2* can control programmed cell death not only by regulation of *SH3RF1* but also via other molecular pathways. The gene expression data obtained in the present study suggested that the *SH3RF2* gene can be a principal factor for controlling exercise-induced apoptosis. Therefore, the next step was to evaluate the possible interactions of detected polymorphisms and racing performance traits.

### Analysis and association of a polymorphism in the SH3RF2 gene and racing results

Based on RNA-seq data, a missense variant ((*SH3RF2*:c.796 T > C:(p.Ser266Pro)) affecting a conserved residue was detected.. Equine SH3RF2 (UniProtKB - F6YV64) contains 4 domains: one RING-type domain and three SH3 (src Homology-3) domains that are responsible for protein-protein interactions in signal transduction pathways [23]. The p.Ser266Pro substitutionis localized in the gene region encoding the protein fragment between the second and third SH3 domain (253 to 382 amino acids). Genesilico Metasever [24] based protein comparisons showed that the *SH3RF2*:c.796 T > C variant possibly affect protein structure and function. The missense variant was associated with the secondary structure of the protein through differences in the alpha helix and beta strand patterns between wild type and mutant proteins (Additional file 3: Figure S2). The amino acid substitution might also modify the protein solvation, as presented by the ratio of the solvent-accessible surface area of the residues observed in the protein structure to that observed in an extended tripeptide (Gly-X-Gly) conformation according to different prediction methods, including two the most common: SPINE [25] and SABLE [26] (Additional file 4: Figure S3). Generally, the differences in both the secondary structure and solvation are critical during three-dimensional protein folding and can affect the final form of the protein and its function [27, 28]. Thus, we hypothesized that the identified c.796 T > C variant influences the function of the SH3RF2 protein and, as a result, determines selected phenotypic traits related to adaptation to exercise. The research performed in chicken and cattle indicated on possible role of SH3RF2 on growth in both species. In chicken, Rubin et al. [29] detected a deletion within *SH3RF2,* which was present at low frequency in a low-growth line and could be the causative mutation for body weight. Moreover, Hanotte et al. [30] showed that bovine *SH3RF2* gene is localized near to the QTL region associated with body weight and was proposed as one of gene located within the selection signature region in French beef cattle [31].

Analysis of the *SH3RF2:*c.796 T > C genotype frequencies showed that the most frequent were horses with *CC* (47.7%) and *TC* (43.7%) genotypes, whereas *TT* homozygotes accounted for 8.6%. Our results showed a significant association of the missense variant in the *SH3RF2* gene and racing results in Arabian horses. The serine substitution by proline at the amino acid 266 position (*CC* genotype) was negatively associated with the probability of winning races, the number of races in which horses occurred and financial benefits obtained in prizes. Horses with the *TT* genotype won significantly more often (*p* < 0.05) in competition that horses with the other genotypes (Table 1). Furthermore, *TT* horses won the most financial benefits, both in regard to total winnings and in winnings per race in which the horses participated. The opposite results were obtained for *CC* homozygote horses and heterozygotes obtained intermediate values (Table 1). The significantly better racing results observed for homozygotes horses with the wildtype T alleles compared to *CC* horses might indicate that amino acid substitution is related to impairment of the function of the SH3RF2 protein. The presented association of the SH3 domain, which contains a ring finger 2 gene, and performance traits is a novel finding in horses and has not been reported in humans or other species to date.

**Table 1:** Association of the identified polymorphism within the *SH3RF2* (*POSHER)* gene and the racing results in Arabian horses

Race traits	*SH3RF2:*c.796 T > C			
		Mean	SE	pval
1st place win	TT	1.24	0.13	a
	TC	0.83	0.11	ab
	CC	0.55	0.33	b
2nd place win	TT	1.11	0.13	
	TC	0.96	0.14	
	CC	0.82	0.35	
3rd place win	TT	1.40	0.12	
	TC	1.03	0.10	
	CC	1.01	0.38	
Total of wins from 1st to 3rd places	TT	4.00	0.30	a
	TC	2.61	0.21	b
	CC	2.56	0.83	b
Numbers of starts per horse	TT	10.2	0.48	a
	TC	7.4	0.49	b
	CC	7.2	1.65	b
Total wins showed in money ($)	TT	2549	254.56	a
	TC	1637	179.08	b
	CC	1178	1802.17	b
Wins showed in money per race ($)	TT	484	107.75	a
	TC	239	120.12	ab
	CC	189	494.00	b

The number of horses in each genotype group – TT *n* = 22; TC *n* = 109; CC *n* = 119; the means with various letters differ at p < 0.05 (a, b).; SE – standard error

Reports from various authors confirmed the important role of both *SH3RF1* and *SH3RF2* in apoptosis [11, 12] and body growth [29, 30]. On the other hand, Kim et al. [32] did not detect a significant role for the *SH3RF2* gene in myoblast differentiation in in vitro studies in quail myoblast cells.

The skeletal muscle *SH3RF2* gene expression profile during a long-term training cycle indicated that this gene is related to exercise adaptation to training and fitness maintenance, probably via regulation of the apoptotic processes. The identified *SH3RF2:*c.796 T > C missense polymorphism was associated with selected racing performance traits, which will be essential information during the evaluation of horse exercise predisposition. In addition, previous reports have shown that performance heritability for flat gallop racing is from 0.15 to 0.55 [33, 34]. This high heritability indicates the possibility of improving the exercise phenotype by genetic selection over a relatively short time period.

## Conclusions

In the present study, the worse racing results obtained for *CC* homozygotes clearly indicated that the c.796 T > C SNP was negatively associated with the function of the SH3RF2 protein. The reported association results and the frequencies of the *TC* and *TT* genotypes suggest the possibility of utilizing this polymorphism in selection to improve racing performance in Arabian horses. Furthermore, future research should establish the potential association of *SH3RH2* with an exercise phenotype, for example sprint, endurance or strength types of predisposition to effort in horses of different breeds. The usage of the identified SNP in selection could allow the improvement of performance features and the maximization of results by the selection of training according to individual horse predisposition.

## Additional files

Additional file 1: **Table S1.** Primer sequence and PCR efficiency of the analysed genes and endogenous controls. (DOCX 13 kb)

Additional file 2: **Figure S1.** The predicted and experimentally determined interactions of the *SH3RF2* - SH3 domain-containing ring finger 2 (String database) for the *Homo sapiens* (A) and *Equus caballus* (B) references. Line colour indicates the predicted mode of action (pink- interactions that were experimentally determined; blue interactions from curated databases; black – co-expression; green – text mining associations and interactions based on relevant publications mentioning a transfer from other organisms). (PNG 1266 kb)

Additional file 3: **Figure S2.** Modification of the secondary structure of the protein: the alpha helix (H) and beta strand (E) patterns in a protein with the wild allele (p.266Ser) and mutant allele (p.266Pro). The figure shows local sub-structures, including the alpha helix and the beta strands, according to different prediction methods, including SPINE and SABLE methods (marked in a red frame). (JPG 149 kb)

Additional file 4: **Figure S3.** Modification of protein solvation presented as the ratio of the solvent-accessible surface area of the residue observed in the protein structure to that observed in an extended tripeptide (Gly-X-Gly) is shown for a protein with the wild allele (p.266Ser) and mutant allele (p.266Pro). An amino acid residue is considered buried (B) if the relative solvent accessibility (RSA) value of the residue is smaller than a specified threshold or exposed (−). The prediction, according to methods including SPINE and SABLE, is marked with a red frame. (JPG 163 kb)

## Abbreviations

GLM: General linear model
RIN: RNA Integrity Number
UTR: Untranslated Region
