# Supplementary material for: Molecular characterization of the apoptosis-related SH3RF1 and SH3RF2 genes and their association with exercise performance in Arabian horses
Source: BMC Vet Res. 2018 Aug 14;14:237. doi: 10.1186/s12917-018-1567-0 (PMC6092840; doi:10.1186/s12917-018-1567-0)
Supplement: Supplementary file 1 — Table S1. Primer sequence and PCR efficiency of the analysed genes and endogenous controls. (DOCX 13 kb) [file 12917_2018_1567_MOESM1_ESM.docx]

| Additional File 1: Table S1. Primer sequence and PCR efficiency of the analysed genes and endogenous controls. | | | | | |
| --- | --- | --- | --- | --- | --- |
| **Gene symbol** | **Gene name** | **References** | **Primers** | **Amplicon length** | **PCR efficiency** |
| *SH3RF1* | SH3 Domain Containing Ring Finger 1 | ENSECAG00000021240 | F GCTTTTTCCCCACCAACTTT  R CAGCCCAGTTTTCATCCACT | 174 | 2.00 |
| *SH3RF2* | SH3 domain containing ring finger 2 | ENSECAG00000024467 | F GACAGCAGGAAAAGCAGGAC  R CTGACTCCGTTGACCTCTCC | 221 | 1.97 |
| *B2M* | beta-2-microglobulin | ENSECAG00000000685 | F TGTCTTTCAGCAAGGACTGG  R CAAGCCTTCATGATGCTGGT | 159 | 1.98 |
| *GAPDH* | glyceraldehyde-3-phosphate dehydrogenase | ENSECAG00000022051 | F TCACCAGGGCTGCTTTTAAC  R GCCTTTCCGTTGATGACAAG | 156 | 2.00 |
