# Supplementary figures and images for: Molecular characterization of the apoptosis-related SH3RF1 and SH3RF2 genes and their association with exercise performance in Arabian horses
Source: BMC Vet Res. 2018 Aug 14;14:237. doi: 10.1186/s12917-018-1567-0 (PMC6092840; doi:10.1186/s12917-018-1567-0)

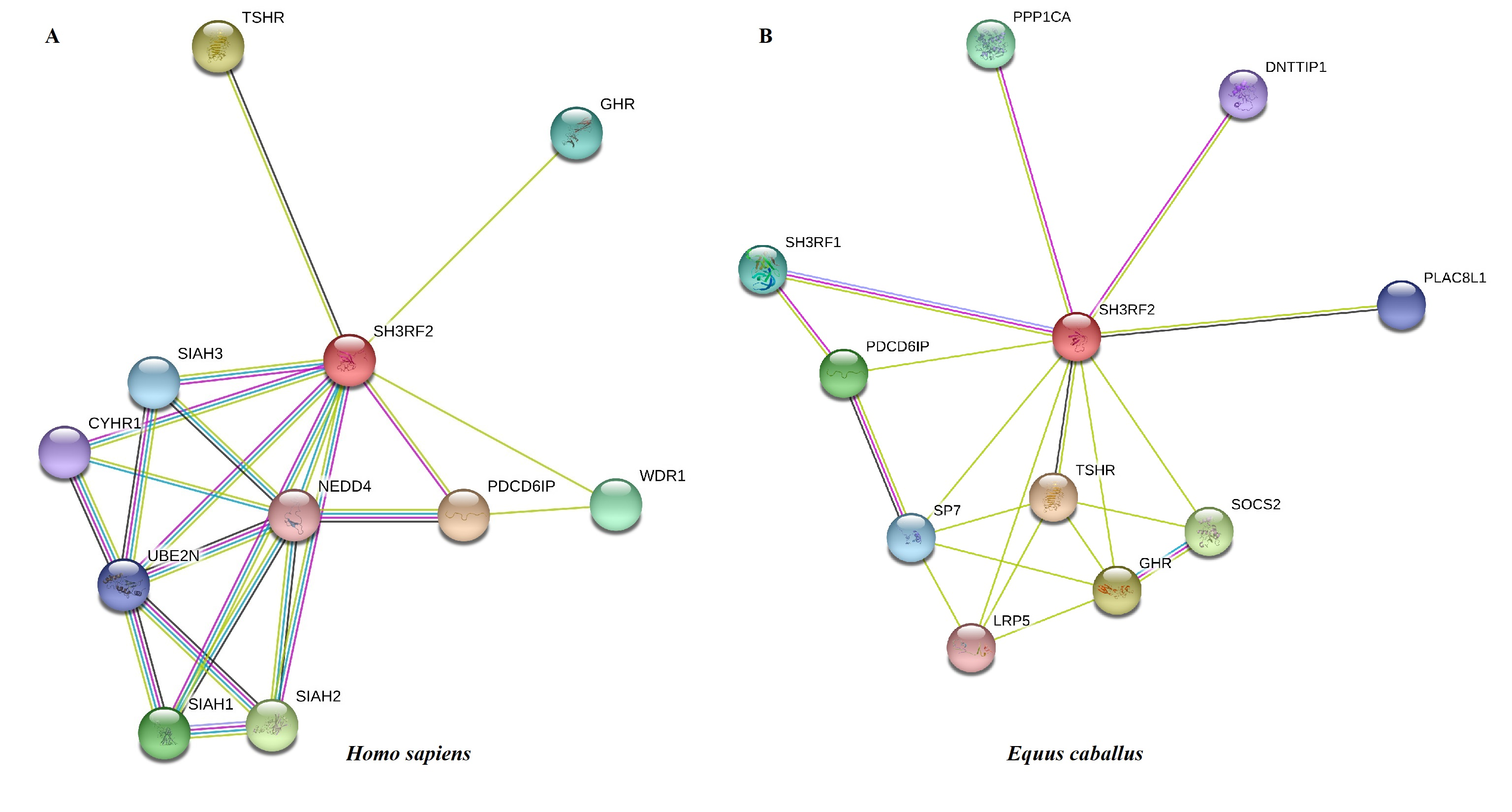

Supplement: Supplementary file 2 — Figure S1. The predicted and experimentally determined interactions of the SH3RF2 - SH3 domain-containing ring finger 2 (String database) for the Homo sapiens (A) and Equus caballus (B) references. Line colour indicates the predicted mode of action (pink- interactions that were experimentally determined; blue interactions from curated databases; black – co-expression; green – text mining associations and interactions based on relevant publications mentioning a transfer from other organisms). (PNG 1266 kb) [file 12917_2018_1567_MOESM2_ESM.png]

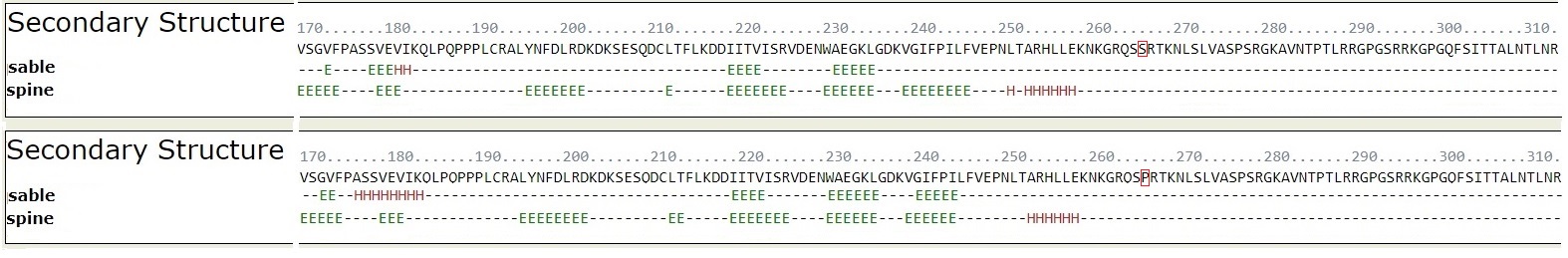

Supplement: Supplementary file 3 — Figure S2. Modification of the secondary structure of the protein: the alpha helix (H) and beta strand (E) patterns in a protein with the wild allele (p.266Ser) and mutant allele (p.266Pro). The figure shows local sub-structures, including the alpha helix and the beta strands, according to different prediction methods, including SPINE and SABLE methods (marked in a red frame). (JPG 149 kb) [file 12917_2018_1567_MOESM3_ESM.jpg]

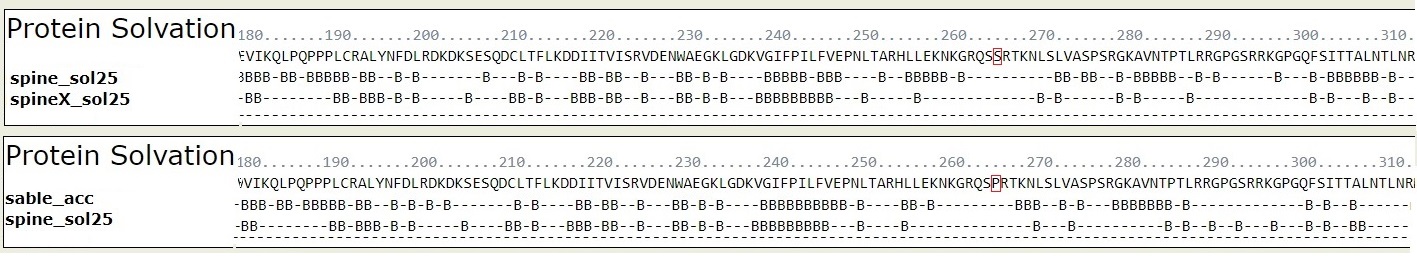

Supplement: Supplementary file 4 — Figure S3. Modification of protein solvation presented as the ratio of the solvent-accessible surface area of the residue observed in the protein structure to that observed in an extended tripeptide (Gly-X-Gly) is shown for a protein with the wild allele (p.266Ser) and mutant allele (p.266Pro). An amino acid residue is considered buried (B) if the relative solvent accessibility (RSA) value of the residue is smaller than a specified threshold or exposed (−). The prediction, according to methods including SPINE and SABLE, is marked with a red frame. (JPG 163 kb) [file 12917_2018_1567_MOESM4_ESM.jpg]
